# Ten years of the Hunter Outcome Survey (HOS): insights, achievements, and lessons learned from a global patient registry

**DOI:** 10.1186/s13023-017-0635-z

**Published:** 2017-05-02

**Authors:** Joseph Muenzer, Simon A. Jones, Anna Tylki-Szymańska, Paul Harmatz, Nancy J. Mendelsohn, Nathalie Guffon, Roberto Giugliani, Barbara K. Burton, Maurizio Scarpa, Michael Beck, Yvonne Jangelind, Elizabeth Hernberg-Stahl, Maria Paabøl Larsen, Tom Pulles, David A. H. Whiteman

**Affiliations:** 1 Department of Pediatrics, University of North Carolina at Chapel Hill, Chapel Hill, NC USA; 2 Willink Unit, Manchester Centre for Genomic Medicine, St Mary’s Hospital, Manchester and Academic Health Sciences Centre, Central Manchester University Hospitals NHS Foundation Trust, Manchester, UK; 3 Department of Pediatric Nutrition and Metabolic Diseases, The Children’s Memorial Health Institute, Warsaw, Poland; 4 UCSF Benioff Children’s Hospital Oakland, Oakland, CA USA; 5 Genomic Medicine Program, Children’s Hospitals and Clinics of Minnesota, Minneapolis, MN USA; 6 Department of Pediatrics, Division of Genetics, University of Minnesota, Minneapolis, MN USA; 7 Centre de Référence des Maladies Héréditaires du Métabolisme, Hôpital Femme Mère Enfant, Bron, France; 8 Department of Genetics/UFRGS and INAGEMP, Medical Genetics Service/HCPA, Porto Alegre, Brazil; 9 Division of Genetics, Birth Defects and Metabolism, Ann & Robert H. Lurie Children’s Hospital of Chicago, Northwestern University, Chicago, IL USA; 10 Rare Disease Centre, Helios Dr Horst Schmidt Clinic, Wiesbaden, Germany; 11 Department of Women’s and Children’s Health, University of Padova, Padova, Italy; 12 Department of Pediatrics, University Medical Center, Johannes Gutenberg University, Mainz, Germany; 13 Jangelind Consulting AB, Stockholm, Sweden; 14 Late Phase Solutions Europe AB, Täby, Sweden; 15 Shire Human Genetic Therapies, Inc., 300 Shire Way HA100-310, Lexington, MA 02421 USA; 16 Shire, Zug, Switzerland; 17 Present address: Shionogi, Inc., Florham Park, NJ USA; 18 Present address: Ultragenyx Pharmaceutical, Inc., Basel, Switzerland

**Keywords:** Patient registry, Mucopolysaccharidosis type II, Hunter syndrome, Enzyme replacement therapy

## Abstract

Mucopolysaccharidosis type II (MPS II; Hunter syndrome; OMIM 309900) is a rare lysosomal storage disease with progressive multisystem manifestations caused by deficient activity of the enzyme iduronate-2-sulfatase. Disease-specific treatment is available in the form of enzyme replacement therapy with intravenous idursulfase (Elaprase®, Shire). Since 2005, the Hunter Outcome Survey (HOS) has collected real-world, long-term data on the safety and effectiveness of this therapy, as well as the natural history of MPS II. Individuals with a confirmed diagnosis of MPS II who are untreated or who are receiving/have received treatment with idursulfase or bone marrow transplant can be enrolled in HOS. A broad range of disease- and treatment-related information is captured in the registry and, over the past decade, data from more than 1000 patients from 124 clinics in 29 countries have been collected. Evidence generated from HOS has helped to improve our understanding of disease progression in both treated and untreated patients and has extended findings from the formal clinical trials of idursulfase. As a long-term, global, observational registry, various challenges relating to data collection, entry, and analysis have been encountered. These have resulted in changes to the HOS database platform, and novel approaches to maximize the value of the information collected will also be needed in the future. The continued evolution of the registry should help to ensure that HOS provides further insights into the burden of the disease and patient care and management in the coming years.

## Background

Randomized controlled clinical trials are the gold standard for assessing the safety and efficacy of new drugs. However, they typically measure only the short-term impact of therapies in limited, strictly controlled patient populations, using standardized treatment protocols [1]. As a result, clinical data obtained in the real-world setting over a longer period of follow-up in larger patient groups are increasingly required to complement and extend findings from clinical trials. This is particularly true for rare diseases, where the number of patients available to participate in trials is often small and the natural disease course may be poorly defined or highly variable [2]. Patient registries are valuable sources of information on disease course and treatment outcomes, and there are now more than 600 rare disease registries in Europe alone [3, 4].

One group of diseases for which patient registries have been particularly valuable is the lysosomal storage diseases (LSD). These are rare, inherited, progressive disorders caused by defects in lysosomal function, and they typically have a highly variable clinical course. Patient registries have been established for a number of LSD, including Gaucher disease, Fabry disease, Pompe disease, and mucopolysaccharidosis types I, II, IV, and VI [5–12], and have provided important insights into the natural history of these conditions and the long-term effects of specific therapies.

Mucopolysaccharidosis type II (MPS II; Hunter syndrome; OMIM 309900) is a rare X-linked disorder caused by deficient activity of iduronate-2-sulfatase (I2S) [13–15] (see Table 1). The disease primarily affects males (estimated incidence, 0.6–1.3 in 100,000 live male births) although a small number of female patients have been described [13, 15, 16]. Historically, management of MPS II was palliative, although two treatment options, enzyme replacement therapy (ERT) and hematopoietic stem cell transplantation (HSCT), are currently available [17, 18]. ERT with recombinant I2S (idursulfase, Elaprase®, Shire, Lexington, MA, USA) became available in the USA in 2006 and in Europe in 2007, and stabilizes many of the somatic signs and symptoms of MPS II [18–22].

**Table 1:** What is mucopolysaccharidosis type II (MPS II)?

• First described in two brothers by Dr Charles A. Hunter in 1917.	
• Caused by deficient activity of the lysosomal enzyme iduronate-2-sulfatase (EC 3.1.6.13), which catalyses a step in the catabolism of the glycosaminoglycans (GAG) dermatan sulfate and heparan sulfate. The accumulation of these in tissues and organs throughout the body contributes to the chronic, progressive, multisystemic manifestations of MPS II [14, 15].	
• The initial clinical signs and symptoms typically emerge within the first few years of life and include recurrent respiratory infections, coarse facial features, joint stiffness, otitis media, hearing loss, umbilical/inguinal hernias, and hepatosplenomegaly [45].	
• The severity of the disease spans a broad range. For clinical purposes, patients are generally considered to fall into one of two categories according to the presence or absence of cognitive impairment. All patients will experience somatic disease manifestations, although progression may be slower in individuals without cognitive impairment [14, 15]. About two-thirds of patients will display progressive central nervous system involvement, initially resulting in learning impairment and abnormal behaviour, followed by the development of profound cognitive impairment [12].	

The Hunter Outcome Survey (HOS) was established in 2005 and the data collected have been used to address post-approval commitments relating to Elaprase. This global, multicentre, longitudinal, observational registry collects real-world data on the clinical presentation and progression of MPS II, and the long-term safety and effectiveness of intravenous ERT with Elaprase. Here, we discuss the contributions that data collected in HOS have made to our knowledge of MPS II and share insights into the nature of this type of clinical study, lessons learned during the past decade, and the new challenges to be overcome in the future.

## Registry design and objectives

HOS is a long-term open-ended global registry designed to collect information on patients with MPS II based on data obtained during routine patient visits and assessments [12]. The registry is open to individuals with a biochemically or genetically confirmed diagnosis of MPS II, including those who are untreated, those who are receiving treatment with Elaprase, and those who have undergone HSCT. Patients receiving ERT with a product other than Elaprase are not eligible for inclusion. Written informed consent is obtained from each patient, their parents, or legal representative. Data from individuals who are alive at HOS entry (prospective patients) can be entered; where local regulations permit, information from those who died before enrollment (historical patients) is also collected.

A broad range of disease- and treatment-related information is captured in the registry (both prospectively and retrospectively), with the primary objective of monitoring the long-term safety and effectiveness of ERT with Elaprase in patients with MPS II (registry endpoints are shown in Table 2). Secondary objectives of HOS are to elucidate the natural history of the disease in untreated patients and to monitor dosing regimens of Elaprase in treated patients. In addition, participating patients and/or their parents can complete the Hunter Syndrome Functional Outcomes for Clinical Understanding Scale (HS-FOCUS) questionnaire, which is used to assess the impact of MPS II on patients’ daily lives. The observational nature of the registry means that there is no predefined sample size and that a hypothesis-free approach is employed. As a result, any data analyses are considered to be exploratory.

**Table 2:** Registry endpoints

Primary endpoints	
Safety of idursulfase • Occurrence of infusion-related reactions and other adverse events (including serious adverse events) • Immunogenicity, as determined by time to first positive antibody response (antibody level and isotype), antibody titre, isotype, and neutralizing antibodies Effectiveness of idursulfase • Urinary glycosaminoglycan levels • Growth parameters (height, weight, head circumference) • Distance walked in the 6-min walk test • Left ventricular mass index (calculated by echocardiography) • Pulmonary function (measured by forced expiratory volume in 1 s and forced vital capacity) • Liver and spleen size (as estimated by palpation) • Prevalence of cardiac- and pulmonary-related hospitalizations • Deaths (including age at death and cause of death)	
Secondary endpoints	
• Evaluation of the natural history of MPS II (based on the following signs and symptoms: hepatosplenomegaly, central nervous system involvement, skeletal involvement, and signs and symptoms of cardiac, pulmonary, and ear, nose, and throat involvement) • Evaluation of dosing of idursulfase (prescribed dose, administered dose, total infusion time, missed infusions, and reasons for missed infusions) • Scoring in five domains in the patient- and parent-reported versions of the HS-FOCUS questionnaire	

*HS-FOCUS* Hunter Syndrome Functional Outcomes for Clinical Understanding Scale, *MPS II* mucopolysaccharidosis type II

**Fig. 1:**
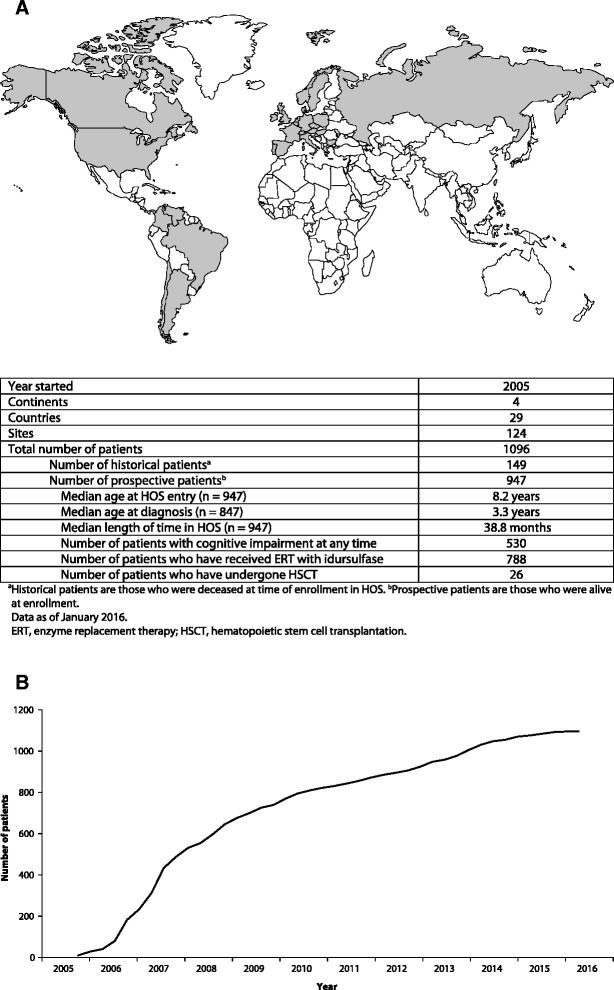
Key facts about the Hunter Outcome Survey. **a** The global reach of HOS. Countries with active HOS sites are indicated in grey. **b** Number of patients enrolled in HOS since 2005

HOS is operational in many countries around the world. The registry has grown significantly over the past 10 years, and as of January 2016, 1096 patients from 124 clinics in 29 countries have been enrolled (Fig. 1). This is a major achievement considering the rarity of MPS II, and HOS is the largest global source of data from patients with the disease. As with all clinical studies, each participating clinic has an Investigator and the registry complies with all relevant regulations and best practices for ethical conduct. Strategic direction and long-term scientific planning are overseen by a Steering Committee, which comprises an international group of physicians with expertise in a range of different disciplines.

## Achievements of HOS over the past decade

### Understanding the clinical presentation and progression of MPS II

**Fig. 2:**
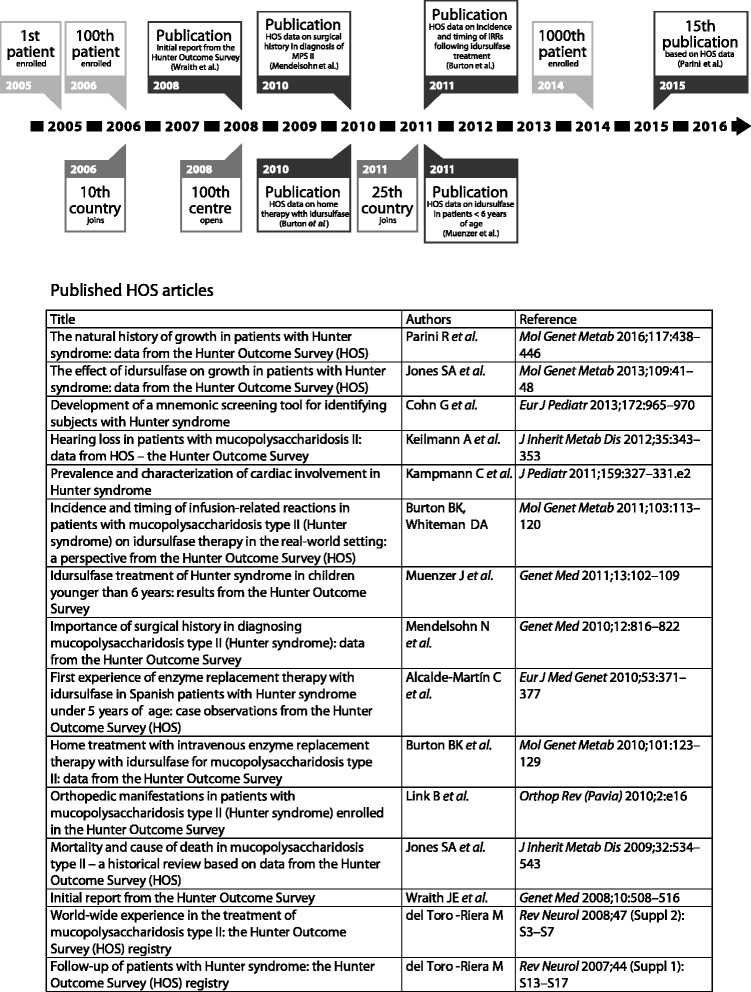
Key landmarks in the history of HOS and associated publications

Analyses of data from the large number of patients in HOS have made a significant contribution to our understanding of the clinical presentation and progression of the disease [12, 23–29] (Fig. 2). For example when the registry was initiated, quantitative data about the timing and prevalence of the clinical manifestations of MPS II were limited. One of the first analyses therefore looked at the time of symptom onset [12]. This demonstrated that the median age of symptom onset was 1.5 years, with otitis media and abdominal hernia the earliest presenting clinical features [12], extending and validating some of the early descriptive reports of patients with MPS II in a larger sample of over 200 patients. This information is particularly valuable because physicians may care for only a small number of individuals with the disease, if any, during their career.

The diagnosis of MPS II is challenging and often delayed because of the non-specific and highly variable nature of the clinical presentation of the disease [30]. An analysis of surgical history in prospective patients in the registry revealed a distinct pattern of procedures that was common in individuals with MPS II [28]. In particular, tympanostomy, inguinal hernia repair, and carpal tunnel release were performed in a greater proportion of the study population than in the general population, and repeat operations for hernia repair and carpal tunnel release were common in patients with MPS II [28]. It is hoped that knowledge of this characteristic pattern of surgical procedures, together with a greater awareness of the early signs and symptoms of the disease among physicians from different medical specialties, will help to facilitate prompt diagnosis and management of patients with MPS II.

### Insights into patient management

The pivotal clinical trials demonstrated that idursulfase was generally well tolerated, with a similar safety profile to that reported for ERT in patients with other MPS [19, 21, 22], and data from HOS have enabled these findings to be extended [31–37]. In particular, an analysis of data from 104 patients treated with idursulfase for at least 1 year demonstrated that, of the 33 patients who had an infusion-related reaction (IRR), the majority (28/33; 85%) experienced their first IRR during the first 3 months of treatment. In addition, most IRRs were mild or moderate in severity and could be managed without interrupting treatment [33]. This information enabled the development of guidance on the management of IRRs [33].

The broad age range of patients enrolled in the registry also allowed an analysis to be performed of safety and preliminary clinical outcomes in patients younger than 6 years of age [37], extending knowledge beyond that obtained from the clinical trials of idursulfase which were restricted to individuals older than 5 years. No new safety issues were identified, and the data suggested that idursulfase has a beneficial effect on palpable liver size [37]. These findings, together with data from subsequent studies in young patients [22], have helped to make idursulfase available to children younger than 6 years old, permitting early initiation of treatment, which is important for patients with this progressive disease [38].

HOS has also provided useful information on the safety and feasibility of home infusions with idursulfase. This option, available in several countries, can help to ease the burden of the disease and its management on patients and their families [39]. A study of 59 individuals who received home therapy for at least 12 months reported that five IRRs occurred in two patients [32]. These IRRs were readily managed at home by either slowing or stopping the infusion and by appropriate pre-treatment with antihistamines. These findings enabled the development of guidelines and an algorithm to facilitate the transition of patients with MPS II from receiving therapy in a clinical setting to receiving infusions at home [32]. The increasing number of treated patients enrolled in the registry should lead to further key insights into the safety and effectiveness of ERT with idursulfase in the future. It should be noted that although patients who have received HSCT are eligible for inclusion in the registry, only a small number have been enrolled (Fig. 1). In addition, the assessment of outcomes in these patients is beyond the scope of HOS and so the safety and effectiveness of HSCT in patients with MPS II have not been assessed using data from the registry.

Finally, it is increasingly recognized in the field of rare diseases that patient-reported outcomes and patient involvement in research are important for facilitating measurement of treatment benefits and quality of life and for ensuring that the care provided is patient centred [1, 3]. The HS-FOCUS questionnaire was developed to obtain insight into the effects of MPS II on the daily lives of patients and their parents and/or caregivers [40, 41]. HS-FOCUS data have been collected in HOS since 2006, and it is hoped that this information will enhance our understanding of the impact of MPS II on the daily lives of patients and their families and potentially lead to improvements in patient care.

## Challenges encountered in HOS

### Data collection and analysis

Although patient registries provide valuable longitudinal data from a large, broad patient population, it is important to take into account the differences between data collected in a formal clinical trial and those collected in a registry. HOS is non-interventional and is designed to acquire ‘real-world’ data from individuals with MPS II during routine clinical practice [12]. As a result, the methods and techniques used for clinical assessments and laboratory assays are not standardized across participating clinics. In addition, the frequency of follow-up visits may vary considerably between patients. It is also important to note that the clinical assessments performed for each patient may differ as a result of variation in disease severity between patients and differences in the standards of care and resources between countries. For example, there is no standard method that is used in all countries for assessing the cognitive and behavioral aspects of MPS II [42].

The multisystem, progressive nature of MPS II also means that each patient typically undergoes numerous clinical and biochemical assessments [43] and is often seen by many different specialists, such as cardiologists, neurologists and otorhinolaryngologists [18, 43]. These specialists are not necessarily directly involved or familiar with HOS, and the specialist clinical assessments and investigations are not always performed at the same site where data entry takes place or at routine visits with the managing physician. The collection of complete, high-quality, uniform data for all patients is therefore often difficult and this can make data analyses particularly challenging. For example, it is not always possible to collect a full set of longitudinal information for each patient. As a result, the number of patients whose data can be used for analysis varies depending on the clinical parameters of interest and the time frame studied. There is also no formal control group and so there are limitations associated with analyses of idursulfase treatment effects. Although a number of untreated patients have been enrolled in the registry, differences in clinical characteristics, the care received, the frequency of clinical visits, and the availability of follow-up data mean that comparisons with treated patients are not straightforward.

Several strategies have been introduced to help participating clinics to increase data completeness. These include the provision of data entry support and improvements to the HOS database platform to make it more user-friendly. A set of key clinical core data variables comprising the minimum data set that should ideally be captured for each patient has also been identified and clinics are asked to focus on collecting these data for each of their patients. In addition, HOS will need to continue to evolve to ensure that the data captured accurately reflect current routine patient care in different countries, as well as new developments in our understanding of MPS II and its management. In the future, it will be important to take advantage of technological advances in database functionality, for example to allow the entry of items such as magnetic resonance imaging scans, and to make sure that the database is updated to capture results from new clinical assessments and tools that are developed in the future. This should help to ensure that the value of the data collected in the registry and the analyses performed are maximized and meet the demands of multiple stakeholders.

### Meeting regulatory requirements

As a condition of the marketing authorization for Elaprase, regulatory agencies in European and other countries (including USA and Canada) required additional long-term safety and effectiveness data in patients with MPS II being treated with idursulfase. Data requirements for specific long-term outcome measures have continued to evolve over time and currently include assessments of pulmonary and cardiovascular morbidity and mortality, urinary glycosaminoglycan levels, and antibody levels.

It is important to note that there are a number of challenges associated with assessing these treatment outcomes. For example, pulmonary function can be assessed using forced vital capacity (FVC) testing and the 6-min walk test (6MWT). However, these tests are unsuitable for particular patient subgroups (e.g. those under 5 years of age and individuals with progressive cognitive impairment) and may not be performed as part of routine patient visits [22, 38, 44]. This makes the evaluation of treatment effects in these patients particularly challenging. The progressive, multisystemic nature of MPS II also means that it is often impossible to collect full, clinically meaningful data sets for many patients, particularly those with advanced disease. Furthermore, baseline data are not available for a substantial number of patients because of the voluntary, non-interventional nature of the registry and because patients may begin treatment many years before HOS entry. As a result, it is difficult to demonstrate long-term treatment effectiveness based on the information available in the database and it is likely that new methods will need to be developed and implemented to address this.

## Conclusions

Building on its primary objective of documenting the long-term safety and effectiveness of idursulfase in patients with MPS II, HOS has provided valuable information on the clinical presentation and progression of the disease, as well as its management. These are considerable achievements, and the registry has provided information on a larger, broader patient population than has been, or could be, studied in formal clinical trials. The multinational nature of HOS and the engagement of experts in MPS II are key strengths of the registry and will be essential to ensure that the registry continues to provide valuable insight into the similarities and differences in patient demographics and management between countries.

A number of challenges will, however, need to be overcome. As further developments are made in the management of patients with this progressive disorder, HOS will need to evolve to ensure that the data collected continue to reflect current practice for patient management accurately and can be used to evaluate the long-term effects of treatment. Patient-reported outcomes are also increasingly being recognized as having an important role in complementing traditional clinical data when assessing the value of treatment in rare diseases [1, 3]; therefore, the continued collection of data from the HS-FOCUS questionnaire remains a priority. Achieving further improvement in the quality and comprehensiveness of the data will remain an important goal, and new initiatives to facilitate this are likely to be required. One approach may be to enable direct data entry by patients and/or their family members, although the feasibility and potential risks of such an approach would need to be considered carefully and discussed with all relevant stakeholders. In addition, it is important to note that idursulfase does not cross the blood–brain barrier and so treatment of neurological aspects of the disease remains challenging. For this reason, several novel approaches are currently being investigated, with the aim of improving the care of patients with MPS II and neurological involvement in the future.

Over the next decade, the implementation of new approaches to aid data collection and entry, and to enhance methods for the evaluation of treatment effects over the long term, should help to ensure that the value of the information collected in HOS is maximized. Ultimately, this will help to ensure that this registry continues to improve our understanding of MPS II and its management in real-world clinical practice.

## Abbreviations

ERT: Enzyme replacement therapy
HOS: Hunter Outcome Survey
HS-FOCUS: Hunter Syndrome Functional Outcomes for Clinical Understanding Scale
HSCT: hematopoietic stem cell transplant
I2S: Iduronate-2-sulfatase
IRR: Infusion-related reaction
MPS II: Mucopolysaccharidosis type II
